# From ERPs to MVPA Using the Amsterdam Decoding and Modeling Toolbox (ADAM)

**DOI:** 10.3389/fnins.2018.00368

**Published:** 2018-07-03

**Authors:** Johannes J. Fahrenfort, Joram van Driel, Simon van Gaal, Christian N. L. Olivers

**Affiliations:** ^1^Department of Experimental and Applied Psychology, Institute Brain and Behavior Amsterdam (iBBA), VU University Amsterdam, Amsterdam, Netherlands; ^2^Department of Psychology, University of Amsterdam, Amsterdam, Netherlands; ^3^Amsterdam Brain and Cognition (ABC), University of Amsterdam, Amsterdam, Netherlands

**Keywords:** MVPA, temporal generalization, decoding, EEG signal processing, time-frequency analysis, ERPs

## Abstract

In recent years, time-resolved multivariate pattern analysis (MVPA) has gained much popularity in the analysis of electroencephalography (EEG) and magnetoencephalography (MEG) data. However, MVPA may appear daunting to those who have been applying traditional analyses using event-related potentials (ERPs) or event-related fields (ERFs). To ease this transition, we recently developed the Amsterdam Decoding and Modeling (ADAM) toolbox in MATLAB. ADAM is an entry-level toolbox that allows a direct comparison of ERP/ERF results to MVPA results using any dataset in standard EEGLAB or Fieldtrip format. The toolbox performs and visualizes multiple-comparison corrected group decoding and forward encoding results in a variety of ways, such as classifier performance across time, temporal generalization (time-by-time) matrices of classifier performance, channel tuning functions (CTFs) and topographical maps of (forward-transformed) classifier weights. All analyses can be performed directly on raw data or can be preceded by a time-frequency decomposition of the data in which case the analyses are performed separately on different frequency bands. The figures ADAM produces are publication-ready. In the current manuscript, we provide a cookbook in which we apply a decoding analysis to a publicly available MEG/EEG dataset involving the perception of famous, non-famous and scrambled faces. The manuscript covers the steps involved in single subject analysis and shows how to perform and visualize a subsequent group-level statistical analysis. The processing pipeline covers computation and visualization of group ERPs, ERP difference waves, as well as MVPA decoding results. It ends with a comparison of the differences and similarities between EEG and MEG decoding results. The manuscript has a level of description that allows application of these analyses to any dataset in EEGLAB or Fieldtrip format.

## 1. Introduction

Since Haxby and colleagues popularized MVPA for functional magnetic resonance imaging (fMRI) (Haxby et al., 2001), multivariate approaches have gained widespread popularity. Initially, MVPA was often used as an abbreviation for multi*voxel* pattern analysis, but in recent years it has become more common to let the acronym denote the generally applicable term multi*variate* pattern analysis. MVPA can refer to a number of related multivariate analytical techniques but is typically used when referring to the practice of characterizing (decoding) the difference between experimental conditions based on the observed patterns of brain responses in those conditions. Curiously, although the multivariate nature of EEG has long been recognized (e.g., Peters et al., 1998; Mitra and Pesaran, 1999), widespread adoption of MVPA to decode experimental conditions using brain activity has been much slower in EEG and MEG research than in fMRI. In recent years however, MVPA decoding approaches have started to gain popularity in EEG and MEG research too. Multivariate analysis in EEG and MEG offers a number of analytical advantages over univariate time-series analysis, such as the ability to look at temporal generalization to characterize neural dynamics over time (King and Dehaene, 2014), the use of representational similarity analysis to map different physiological measures or anatomical substrates onto each other (Kriegeskorte et al., 2008; Cichy et al., 2014), as well as the ability to establish a common performance measure to map behavioral onto neural data (Fahrenfort et al., 2017b). Moreover, MVPA allows one to quantify experimental effects without a-priori electrode or channel selection, potentially identifying differences between conditions that are harder to detect using conventional analyses (Fahrenfort et al., 2017a). Indeed, many researchers now prefer to use multivariate analyses over traditional ERP/ERF analyses based on signals averaged over epochs (Mostert et al., 2015; Kaiser et al., 2016; Wardle et al., 2016; Contini et al., 2017; Marti and Dehaene, 2017; Turner et al., 2017).

Consequently, some who have been employing traditional univariate ERP analyses may be considering switching to MVPA or extending their analysis pipelines with MVPA. However, although a number of decoding toolboxes exist, this step can appear daunting to those who have been using software packages with graphical user interfaces (GUIs), like EEGLAB or BrainVision Analyzer. For this reason, we developed the ADAM toolbox (from here on simply referred to as ADAM) for the MATLAB platform. ADAM takes EEGLAB or Fieldtrip data formats as input, and performs multivariate analysis using a relatively simple specification of the required parameters. Although ADAM has no GUI, the toolbox requires no programming experience, only rudimentary knowledge of MATLAB such as opening and closing of text files and running commands in the Command Window. ADAM performs standard analysis of raw EEG/MEG data (both ERP averages and decoding results), but also provides a number of additional capabilities. For example, it is able to compute temporal generalization matrices (King and Dehaene, 2014) and it can run a time-frequency analysis prior to decoding. In this case, results are plotted in a time-by-frequency matrix, or temporal generalization matrices for particular frequency bands. Time-frequency analysis can either be based on total power, or on induced power. Furthermore, ADAM can simultaneously run a forward encoding model (FEM) in addition to a backward decoding model (BDM), allowing one to reconstruct patterns of neural activity that were never present during model generation (Brouwer and Heeger, 2009; Fahrenfort et al., 2017a).

The current article does not cover all of these options, but rather takes a subset of them as an entry-level introduction for those who have been doing ERP research and want to explore multivariate analysis. It covers decoding of raw EEG/MEG data and describes an analysis pipeline in which ERPs are compared to decoding results. It also shows how to compute and visualize temporal generalization matrices which allow one to look at the stability of patterns of neural activity over time (King and Dehaene, 2014). Finally, the analysis pipeline compares decoding of EEG to decoding of MEG data. Note that this article is not primarily intended as an explanation of *why* to perform multivariate decoding analyses (although some advantages of MVPA over ERPs are highlighted), but rather to explain *how* to perform these analyses. At the end of the article, one should be able to run decoding analyses on any epoched EEG dataset in EEGLAB or Fieldtrip format. Along the way, the article briefly explains basic terminology such as decoding, classes/classification/classifier, temporal generalization, train/test schemes (such as k-fold) in the context in which they are first introduced. For more detailed explanations we refer to introductory texts such as Blankertz et al. (2011), King and Dehaene (2014), and Grootswagers et al. (2017). The article also assumes working MATLAB knowledge. Programming experience is not required, but the reader should be able to open and close files in MATLAB and know how to execute snippets of code in the MATLAB Command window, which is easy to learn even for those who have not used MATLAB before.

The data that we analyze in this manuscript come from a publicly available MEG/EEG/fMRI dataset. This dataset contains event-related responses to famous, non-famous and scrambled face stimuli, and was acquired and made available by Daniel Wakeman and Richard Henson (Wakeman and Henson, 2015). The dataset contains the type of factorial design that is common to many experiments. The manuscript is organized as follows: the methods describe where the sample data can be obtained, where to obtain the toolbox and its dependencies, how to install the toolbox on MATLAB and provides code that shows how to run the first level (single subject) analyses. The results section provides the code to run and plot the results from the group analyses. Although somewhat unorthodox, providing these together in the results section improves coherence. This way, the code that generates the plots can be presented together with the plots themselves. The results section contains group analyses of ERPs, group analyses of decoding results, examples to plot forward transformed classifier weights (equivalent to univariate topomaps, which are interpretable as neural sources) (Haufe et al., 2014), and shows temporal generalization matrices of the EEG and MEG results. It also provides an example of how to plot temporal generalization for a specific time window and ends with a direct comparison of EEG to MEG. The discussion considers the degree to which MVPA analyses can provide extra information over standard univariate analysis, based on the results that were presented.

## 2. Materials and methods

### 2.1. Data

The raw data are available at https://openfmri.org/dataset/ds000117. However, due to the size of the original data files in the public repository (which also include fMRI), we have created a slim-sized version of the data in standard Fieldtrip format to facilitate easy reproduction of the analyses as described in this article. These data files can be found on the open science framework by following the following link: https://osf.io/p2k97/files. To replicate the analyses described here, store all files under DATA (20 GB) in a local directory. No pre-processing was applied to the original data other than down-sampling from 1,100 to 275 Hz, and epoching around the target stimuli with an interval between −0.5 and 1.5 s. The MEG data were obtained from an Elekta MEG system, and were processed with MaxFilter 2.2 (Elekta Neuromag) by the original authors (Wakeman and Henson, 2015). To further reduce data overhead, we removed the magnetometers from the original data. Magnetometers have a more diffuse spatial profile with large overlaps between neighboring sensors when compared to planar gradiometers (Gross et al., 2013). Removal of magnetometers after application of a MaxFilter is not uncommon (e.g., see Kloosterman et al., 2015), and a pilot analysis confirmed that this did not substantially affect classification performance. The abovementioned repository includes a MATLAB script under SCRIPTS, that converts the original data as supplied by Wakeman and Henson to the files we posted, but since this step is idiosyncratic to whatever system is used to acquire EEG or MEG data, we did not make it part of the analysis pipeline we describe in the remainder of the text.

### 2.2. Task

The task employed during the experiment was described in detail by Wakeman and Henson (2015). For ease of reference, we briefly explain the task here. Every trial started with a pre-stimulus period between 400 and 600 ms (randomly jittered) containing a white fixation cross on a black background. Next the target stimulus appeared for a random period between 800 and 1,000 ms. The target stimulus was a cut-out of a photo of a face on a black background, overlaid with a white fixation cross. The face could be either a famous, non-famous, or phase scrambled face. Each image was presented twice, with the second presentation occurring either immediately after the previous one (Immediate Repeats), or after 5–15 intervening stimuli (Delayed Repeats). Each type of repeats occurred in 50% of the trials. Face identity was not task relevant, subjects only had to indicate whether a given stimulus was more or less symmetrical than the average amount of symmetry across all photos. Participants used their left or right index finger to indicate symmetry, counterbalanced across subjects.

### 2.3. Participants

Data was collected from 19 participants (8 female). Further details can be found in Wakeman and Henson (2015).

### 2.4. Requirements

ADAM works under a relatively recent version of MATLAB (≥R2012b, older versions might or might not work) with the Signal Processing Toolbox and Statistics Toolbox installed. Further, when running first level (single subject) analyses it depends on a recent version of EEGLAB (Delorme and Makeig, 2004) (≥13, older versions might or might not work) and a recent install of Fieldtrip to perform time-frequency analysis prior to decoding (Oostenveld et al., 2011) (≥2015, older versions might or might not work). Finally, a reasonably modern desktop or laptop computer with standard specifications. More is better (especially RAM), but any computer used for office work should in principle be sufficient. All analyses presented here (three EEG and three MEG comparisons) were executed on a 2013 Macbook Pro with 8GB of memory, using Matlab R2014b, EEGLAB v14_1_1b and Fieldtrip v20170704. The first-level analyses took about 10 h to complete. If one wants to replicate these analyses in a shorter timeframe, it is easy to shorten computation time by lowering cfg.nfolds to 2 instead of 5, which affects the number of folds in the experiment (reducing computation time by 60% to about 4 h). The concept of folds is explained in section 2.9.6 below. Another way to reduce computation time is by lowering the number of subjects in cfg.filenames, e.g., from 19 to 10 (another 50% reduction). Both these changes can be made in the first-level script in section 2.9, and will have little effect on the qualitative patterns of single-subject and group-level results, although some effects may not reach significance. Group-level analyses take very little time and can be executed on the fly.

### 2.5. ADAM toolbox

When replicating the analyses in this article, we recommend to download version 1.0.4 of the ADAM toolbox from Github at https://github.com/fahrenfort/ADAM/archive/1.0.4.zip. This is the version of the toolbox that was used to perform the analyses and generate the figures in this article and is therefore guaranteed to work with the scripts that are provided herein. We also provide version 1.0.4 of the toolbox along with a version of EEGLAB and Fieldtrip that are guaranteed to work with the toolbox under TOOLBOXES on the Open Science Framework here: https://osf.io/8vby7/download.

For regular use of the ADAM toolbox, we recommend to download the latest version of the toolbox by going through http://www.fahrenfort.com/ADAM.htm where users can leave their e-mail before being forwarded to the download site on Github. Keeping track of e-mail addresses allows us to contact users if major bugs come to light. A simulated validation dataset is currently being developed and will in the near future be used to continuously validate core functionality of the toolbox.

### 2.6. Installing

Installing the toolbox and its dependencies is easy. To replicate the analyses in this article, download the file from the repository above and unzip it. This should create a folder called ‘TOOLBOXES' containing all three toolboxes (ADAM, EEGLAB and Fieldtrip). This folder can be placed anywhere (e.g., 'C: TOOLBOXES' on Windows PC, or '/Users/JJF/TOOLBOXES' on a Mac) but do take note of the location. Next, follow the install instructions in the text file “install_instructions.txt” that is contained in that directory. Following these instructions will make sure that MATLAB knows how to find the toolboxes. If all goes well, the following should be displayed in the Command Window (along with some other messages):

FIELDTRIP IS ALIVE

EEGLAB IS ALIVE

ADAM IS ALIVE

When these messages are displayed, all code provided in this article should run smoothly.

### 2.7. ADAM architecture and core functionality

**Figure 1:**
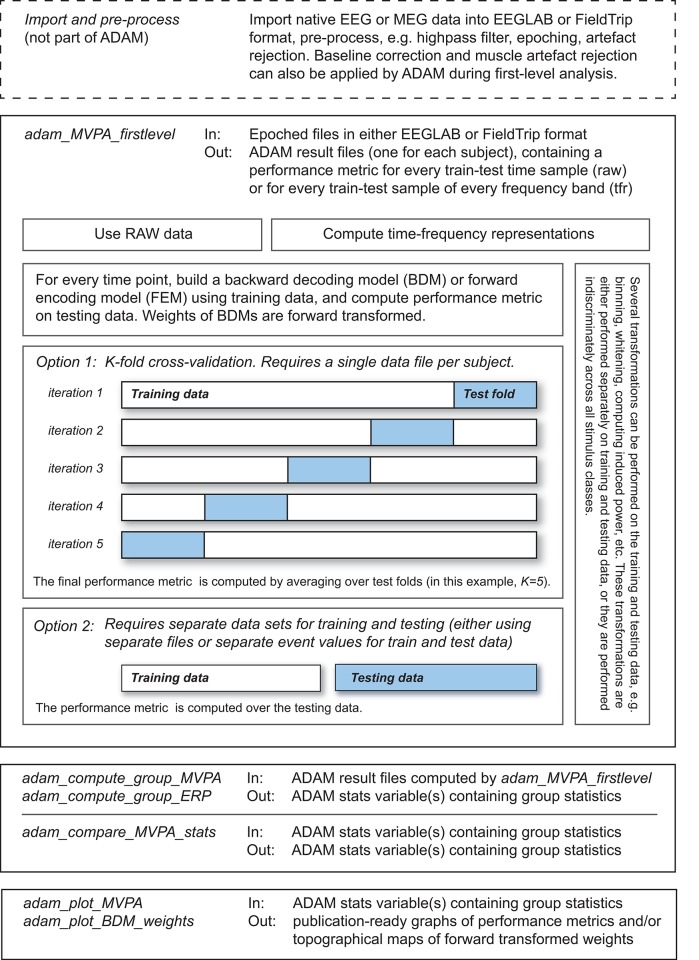
ADAM processing pipeline, from top to bottom. The top left corner of each box states the ADAM function that performs the transformations that are described in the box. The top right describes the input-output transformation that the function performs. The output of each function serves as input for the function described in the box below it. The adam_MVPA_firstlevel box contains more detailed information about train-test algorithms. Further details about functionality and how to execute functions are provided in the main text.

The ADAM processing pipeline is depicted in Figure 1 (from top to bottom). It involves: (1) Data-import and pre-processing (2) first-level single-subject analysis (3) computing group-level statistics and (4) visualization (plotting) of group statistics. These steps are implemented by a number of main ADAM user functions, all starting with the prefix adam_ (also mentioned in the top left corner of each box in Figure 1):

- adam_MVPA_firstlevel (computes and stores first level / single subject results)
- adam_compute_group_ERP (reads single subject ERPs and computes group statistics which can be plotted using adam_plot_MVPA)
- adam_compute_group_MVPA (reads single subject classification performance and computes group statistics which can be plotted using adam_plot_MVPA)
- adam_compare_MVPA_stats (compares outcomes of group analyses, which can be plotted using adam_plot_MVPA)
- adam_plot_MVPA (plots the outcome of the adam_compute_group_ or the adam_compare_MVPA_stats functions)
- adam_plot_BDM_weights (plots topomaps of the classifier weights or forward transformed weights, the latter of which are equivalent to univariate difference maps and are interpretable as neural sources, see (Haufe et al., 2014).

All ADAM user functions can be called from the MATLAB Command Window using the same syntax: result = adam_somefunction(cfg, *input*);

In this expression, cfg (short for configuration) is a variable that specifies the parameters that the function needs. The concept of a cfg variable was borrowed from the Fieldtrip toolbox (Oostenveld et al., 2011), but ADAM is not part of Fieldtrip so their functionalities should not be confused. The *input* variable is not always required. It can either be a variable that contains data, or it can specify a file path to the data. In the remainder of the methods section, we will outline how to use each of the main ADAM functions illustrated in Figure 1 to run a first-level analysis, and how to run and visualize a subsequent group analysis. We will use the Wakeman and Henson dataset (Wakeman and Henson, 2015) as an illustration of how to use the cfg variable to specify analysis and/or plotting parameters at each step of the way.

### 2.8. Data structure

We recommend to use a standard folder structure when analyzing experiments using ADAM: at the highest level a container folder for the experiment that is analyzed. Inside that folder, there should be at least three subfolders: (1) DATA: a folder with EEG/MEG input files, such as the epoched EEGLAB or Fieldtrip files that are downloaded from the repository, or the processed EEG/MEG datafiles of a different experiment. (2) SCRIPTS: a folder that contains MATLAB scripts that perform ADAM analyses that are particular to the experiment. Scripts are snippets of code that tell ADAM how to analyse the data (which are distinct from the ADAM toolbox, so one should not put these scripts inside the toolboxes folder). When saving analysis scripts, it is good practice to prepend the names of these scripts with a canonical prefix so they can be easily recognized as scripts (e.g., prepend all scripts with “run_”), such as run_preprocessing.m, or run_RAW_level1.m. It also helps to add further keywords like “RAW” to indicate that the file contains a script to perform a decoding analysis of raw EEG data, or to use a keyword like TFR to indicate it performs a decoding analysis on time-frequency data. Adding a keyword like “level1” can be used to indicate that the script performs an analysis of the single subject data. Example scripts for the analyses that are described in this article are provided in the text but can also be found in the SCRIPTS folder located at https://osf.io/p2k97/files. (3) RESULTS: a folder that contains the outcome of the single subject analyses (these are often referred to as “first level” analyses), for example when classifying from the EEG whether subjects are viewing faces or scrambled faces, or when classifying whether they were viewing famous faces or non-famous faces. Each such analysis will be stored in a separate folder. This folder in turn will contain deeper levels created by ADAM, reflecting electrode selections and/or specific frequencies on which the analysis was performed, and finally a results file for every subject.

### 2.9. First level (single subject) analysis

In this manuscript, we describe how to run the first level analyses for three main comparisons, using ADAM:

- non-famous faces vs. scrambled faces
- famous faces vs. scrambled faces
- famous faces vs. non-famous faces

These are performed separately on the EEG and MEG dataset, so six analyses in total. The first script we provide below executes the first of these analyses: non-famous vs. scrambled faces of the EEG data. The script starts by specifying some initial variables (such as the names of the input files and the event codes that belong to the various factors/levels in the experimental design, which are needed to run first-level analyses), and subsequently specifies the cfg parameter settings that determine the settings during a first level analysis. Note that in MATLAB notation, comments are preceded by a percent (%) sign and drawn in green. These comments are used to provide a brief explanation of what a particular line of code does but are not actually executed by MATLAB when the code runs. The last line of the script executes the actual first level analysis using the adam_MVPA_firstlevel function, which computes single subject decoding and/or forward encoding results. ERPs are computed automatically when running adam_MVPA_firstlevel. The outcomes of the single subject analyses are stored as files inside the RESULTS folder, which are subsequently read in during group analysis (see section 2.11). This script can also be found in the SCRIPTS folder on https://osf.io/p2k97/files in the file run_firstlevels.m.

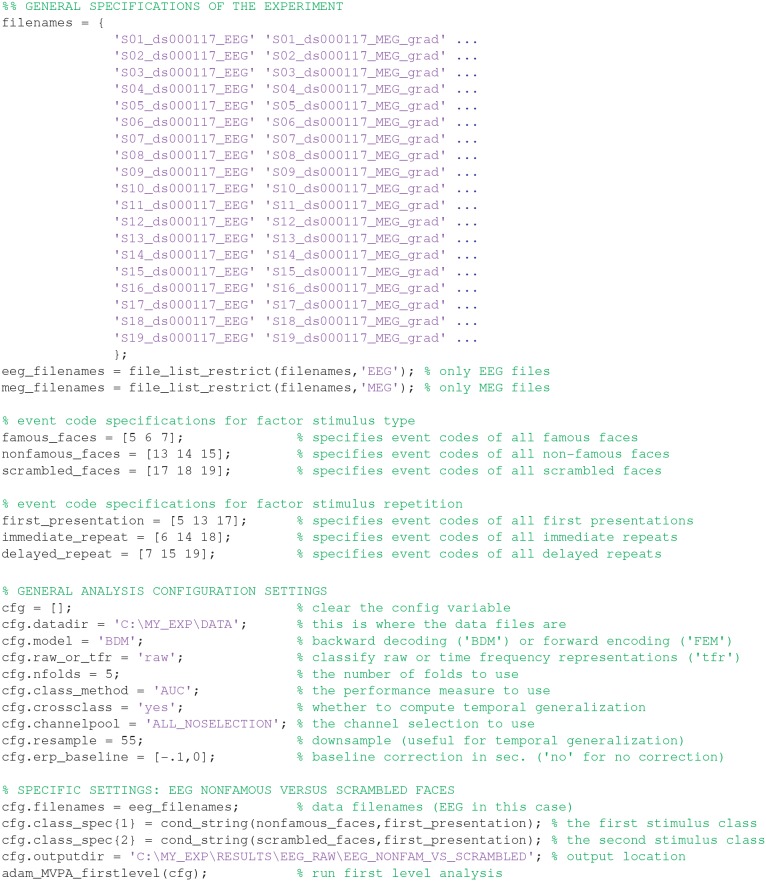



The script above can simply be copied and pasted/executed directly in the MATLAB Command Window or executed from within a MATLAB Editor window by clicking “Run” in the Editor toolbar. The next sections provide a by step explanation of the script by explaining the parameter settings that are used to run the first level analysis using adam_MVPA_firstlevel.

#### 2.9.1. Input filenames

The filenames used for the first level analyses are specified using cfg.filenames, the path to these files is specified in cfg.datadir (see example code above). The toolbox is able to work with two file formats: (1) Standard EEGLAB format with “.set” and “.fdt” extensions (Delorme and Makeig, 2004). (2) A standard Fieldtrip struct saved with “.mat” extension (Oostenveld et al., 2011) either in timelock (ERP/ERF) or non-timelock epoched format. All files should be epoched and the event code that specifies the relevant conditions for analysis needs to be numeric and placed at 0 ms in the epoch (for EEGLAB format) or be contained in a trialinfo field with an event code for each trial (for Fieldtrip format).

Both EEGLAB and Fieldtrip have a large number of importing options for many available EEG/MEG data acquisition formats. The file name specification for the ADAM analysis should list all files in a cell array as in the example code above. Do not use extensions in the filename specification. The toolbox will first attempt to locate “.set” (EEGLAB) files, if it cannot find those it will look for “.mat” files containing a Fieldtrip struct. The function file_list_restrict selects files from the full file list based on a part of the file name. This can be useful in cases like this, where separate EEG and MEG files exist, or when files come from different experimental sessions that need to be analyzed separately etc. The example code above creates a separate array of the EEG files and of the MEG files to be able to run separate first level analyses for EEG and MEG.

It is also possible to train the classifier on one input file and test on another input file by separating the two files using a semi-colon (see sections 2.9.3 and 2.9.6 for more information about train-test schemes). That way one can train a classifier on one task, and test on another, or one can even train the classifier on one subject and test on another subject, as long as both files have the same data format (same number of electrodes etcetera).

#### 2.9.2. Class specifications and balancing

A decoding analysis tries to discriminate between a fixed set of experimental variables using brain data. The algorithm that performs classification is called the “classifier,” and the experimental conditions it tries to discriminate are called the “classes.” Table 1 shows the factorial design of the experiment that is analyzed in the current manuscript. The numbers in the table are the event codes that were used to denote the various events/conditions in the experiment. It is easy to draw a similar table for most experimental designs.

**Table 1:** Factorial design of the experiment, numbers denote event codes.

		**Factor** “**STIMULUS TYPE”**		
		**Famous**	**Non-famous**	**Scrambled**
**Factor**	**First presentation**	5	13	17
**“STIMULUS**	**Immediate repeat**	6	14	18
**REPETITION”**	**Delayed repeat**	7	15	19

Once the event codes in the levels of the factors in the design are assigned to variables (see the code on the previous page), it is easy to set up a class definition, which specifies the conditions or groups of conditions (classes) that the analysis should try to discriminate (classify). For example, to compare famous faces to non-famous faces simply write:

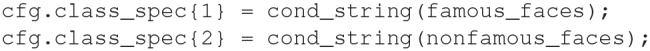



cond_string is an ADAM function that creates string specifications from numbers because ADAM requires string inputs. Thus, the above class definition is effectively the same as:

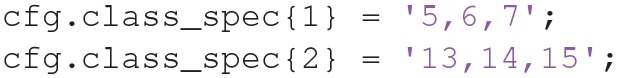



By default, ADAM enforces balanced designs. A design is balanced when the trial counts in the different cells of the factorial design (as in Table 1) are equal. Unbalanced designs (asymmetrical trial counts) can have a number of unintended effects on the type of conclusion that can be drawn from the analysis. There are two types of imbalances: *within class* imbalances and *between class* imbalances. *Within class* imbalances occur when event counts within classes are unequally distributed. For example, this occurs if a decoding analysis compares famous faces to non-famous faces (irrespective of the factor stimulus repetition), while at the same time the design contains many more first presentations than immediate or delayed repeats. In such a case, the outcome might be driven more strongly (or even entirely) by the first presentations than by the repeated presentations. This would impact how generalizable the effect of being famous is across the experimental design. Because designs are often unbalanced, ADAM rebalances designs by applying two types of corrections: within class *undersampling* (throwing out trials) and between class *oversampling* (duplicating trials). The act of rebalancing unbalanced designs through under- or oversampling has been shown to convey clear performance benefits for linear discriminant analysis and area under the curve (Xue and Hall, 2015), which are the classification algorithm and default performance metric that ADAM uses (see sections 2.9.3 and 2.9.5).

*Between class* imbalances occur when an entire class is overrepresented in the analysis. An example of such an imbalance would be when performing decoding of famous faces and non-famous faces, while many more famous faces than non-famous faces exist in the dataset. In such cases the classifier can develop a bias by classifying the majority (or even all) trials as famous faces. Classification performance across trials would be higher than chance even if the classifier has in fact no ability to discriminate famous faces from non-famous faces, due to the simple fact that the majority of trials contain famous faces. Therefore, ADAM rebalances classes by default by making use of a special case of *oversampling* (duplicating trials) in the training set. This is achieved by synthetically generating instances (trials) of the class that has the fewest number of trials (i.e. the minority class). Class instances are generated using a modification of the ADASYN algorithm, which generates instances that maximally drive learning during the training phase (see sections 2.9.3 and 2.9.6) (i.e. by generating synthetic trials that are close to the classifier decision boundary) (He et al., 2008). In the example above, if the class of famous faces contains 150 trials and the class of non-famous faces contains 75 trials, ADAM would generate another 75 synthetic trials of the non-famous faces class so that there are an equal number of trials of both classes in the training set.

Within classes, ADAM applies *event balancing* by default through *undersampling* so that all event types contribute equally to a stimulus class. In the example above, if there are 200 first presentations of a famous face (event code 5), but only 50 immediate repeats (event code 6) and 50 delayed repeats (event code 7) of famous faces, ADAM lowers the trial count of the first presentations to match with the others (so the 200 first presentations would be lowered by randomly selecting 50 of those, to match with the immediate and delayed repeats). It is important to be aware of this, as one may lose a lot of trials if the experimental design is heavily unbalanced within classes. ADAM also allows one to specify an idiosyncratic ratio of each trial type in the class definition. For example, to specify two first presentations for every immediate and delayed repeat, use:

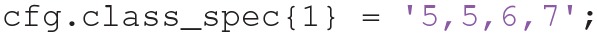



To keep things simple, the analyses that are covered in this article will only classify the first presentation of each stimulus type. The cond_string function makes it easy to create such class definitions by combining levels in the factorial design, as was done in the example code provided. It is also possible to use different class definitions for training and testing, by separating the two using a semi-colon (see sections 2.9.3 and 2.9.6 for more information about train-test procedures).

#### 2.9.3. Model selection

ADAM is able to run two basic models: a backward decoding model (BDM, default) and/or a forward encoding model (FEM, sometimes also referred to as an inverted encoding model) (Brouwer and Heeger, 2009). BDMs allow one to predict an experimental variable (condition) given an observed pattern of brain activity. The experimental variables that the model attempts to discriminate based on brain data are called the classes (see section 2.9.2). The model that makes these predictions is often referred to as the classifier. The process of making class predictions is often referred to as “classification” or “decoding,” and involves a procedure in which some data is first used to train the classifier (build the model), and a set of independent data is used to evaluate its performance (see sections 2.9.5 and 2.9.6). By default, the BDM in ADAM employs Linear Discriminant Analysis (LDA) to perform decoding, a standard decoding algorithm that has been shown to perform well compared to other algorithms (Grootswagers et al., 2017), and which is able to solve classification problems for two or more classes. All analyses described in the current manuscript use a BDM.

While BDMs employ a categorical relationship between brain data and experimental variables, FEMs describe an invertible continuous relationship between experimental variables and brain data, allowing one to predict patterns of brain activity for arbitrary values of the experimental variable (and vice versa). FEMs are most useful when the relationship between the experimental variable and neural activity is continuous (e.g., color, orientation of a bar, position on a circle). It determines the relationship between such a continuous experimental variable and multivariate brain signals using a Channel Tuning Function (CTF). The CTF allows one to reconstruct patterns of neural activity for stimuli that were never used during model generation and vice versa (Brouwer and Heeger, 2009; Fahrenfort et al., 2017a). FEMs too make use of cross-validation, in which independent datasets are used for fitting the model (training) and validating the model (testing), also see section 2.9.6. FEMs are not relevant to the experimental design of the data that are analyzed and presented here, and therefore outside the scope of this manuscript. However, there is considerable literature available for those who want to know more (Foster et al., 2016, 2017). The cfg.model parameter allows one to specify whether ADAM should run a BDM or a FEM during analysis.

#### 2.9.4. Raw or time-frequency

ADAM is able to either perform MVPA analyses on raw EEG/MEG data, or first perform a time-frequency decomposition into frequency bands prior to analysis. In the current manuscript, we only analyze raw data, but ADAM is able to first compute time-frequency representations (TFRs) prior to a BDM or FEM analysis. The cfg.raw_or_tfr parameter specifies whether ADAM should analyze the raw EEG/MEG amplitude over time, or whether it should first compute TFRs by respectively specifying “raw” or “tfr.” It is good practice to store the results from analyses on raw data in a different folder from analyses that are performed on TFR data. When computing TFRs, it is important to realize that the input files for ADAM are always raw data, ADAM will compute the TFRs internally during analysis using Fieldtrip. There are a number of additional options available for TFRs, such as computing induced rather than total power (Klimesch et al., 1998; Pfurtscheller and da Silva, 1999; Fahrenfort et al., 2012). When performing decoding on TFR data, ADAM computes accuracy in a time-by-frequency plot by default, but it can also compute temporal generalization matrices for specific frequency bands when cfg.crossclass is set to 'yes' (see section 2.9.7).

#### 2.9.5. Performance measures

The performance of a classifier quantifies how accurately it can predict class membership based on measured brain activity. There are many conceivable classifier performance metrics, depending on the research question and goal of the analysis. An often-used performance measure in the literature is “accuracy,” the number of correct classifications averaged across all class instances. When ADAM computes accuracy, it does so for each class separately, and then averages across classes (balanced accuracy). For example, when an analysis targets a classification of faces and scrambled faces, ADAM computes accuracy as:

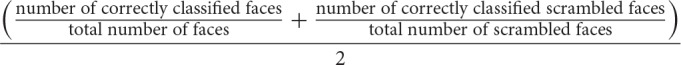



This measure should theoretically produce chance accuracy even when the classifier develops a bias and/or when a stimulus class is overrepresented in the data.

A more sensitive measure to compute classifier performance is Area Under the Curve (AUC) (Bradley, 1997). AUC is the default performance measure that ADAM computes. AUC refers to the area under the receiver operating characteristic, a metric derived from signal detection theory (Wickens et al., 2002). It constitutes the total area covered when plotting the cumulative true positive rates against the cumulative false positive rates for a given classification problem and—like balanced accuracy—is insensitive to classifier bias. In a two-class decoding analysis, this is achieved by plotting the cumulative probabilities that the classifier assigns to instances as coming from the same stimulus class (true positives) against the cumulative probabilities that the classifier assigns to instances that come from the other stimulus class (false positives). AUC takes into account the degree of confidence (distance from the decision boundary) that the classifier has about class membership of individual instances, rather than averaging across binary decisions about class membership of individual instances (as happens when computing standard accuracy). In other words, low confidence decisions contribute less to the AUC than instances about which the classifier is very confident, whereas for accuracy all classifications are treated equally. When ADAM computes AUC in multi-class problems, it uses the average AUC across all pairwise comparisons between classes (Hand and Till, 2001). Therefore, chance AUC performance is always 0.5, regardless of the number of classes that the analysis attempts to discriminate. The performance measure that ADAM should compute can be specified using the class_method parameter, e.g., cfg.class_method = 'AUC'. ADAM can also compute a number of other measures derived from signal detection theory, such, d' ('dprime'), hit rate ('hr') and false alarm rate ('far').

#### 2.9.6. Train-test procedures, K-fold cross validation

A classification analysis usually consists of two steps: one in which a model is fitted to the data (training), and one in which the performance of the model is evaluated (testing). These two steps are usually performed on independent data. If they would be performed on the same data, the performance of the model would not only reflect true differences between stimulus classes, but also differences that occur because of coincidental (noise related) differences between stimulus classes (this is also called “overfitting”). To prevent overfitting from inflating the performance of the model, separate data are used for training the model and testing the model. There are two ways of achieving this goal in ADAM: (1) use two independent data sets, one for training and one for testing or (2) use a single dataset for training and testing using k-fold cross validation. In k-fold cross validation, the trials are split up into k equally sized folds, training on k-1 folds, and testing on the remaining fold that was not used for training. Therefore, the training set is independent from the testing set on that iteration. This procedure is repeated k times until each fold (all data) has been tested exactly once, while on any given iteration the trials used for training are independent from the trials that were used for testing. A graphical illustration of this procedure can be found in Figure 1 in the box that says adam_MVPA_firstlevel. Next, the performance measures obtained at each iteration/fold are averaged to obtain a single performance metric per time point.

In ADAM, the number of folds is specified using the cfg.nfolds parameter. For example, if nfolds is 4, the classifier will train on 75% of the data and test 25% of the data, repeating the process until all data has been tested once. If nfolds is higher than the number of trials in the dataset, ADAM automatically lowers nfolds to a number that implements leave-one-out testing, in which the classifier is trained on all but one trial and then tested on the remaining trial. This would be very time consuming, as the entire process is then repeated equally often as there are trials in the data set. When train and test data are already independent (for example when using different input files for training and testing, or when using different event codes for training and testing), nfolds is disregarded.

#### 2.9.7. Temporal generalization using classification across time

ADAM is also able to cross-classify across time. In this case, the classifier is not only trained and tested on the same point in the trial, but every train time point is also tested on all other time points in the trial. This results in a train × test time performance matrix, also called a temporal generalization matrix. If classifier performance for any given train time point is high when testing on other time points, this means that the pattern that was used to train the classifier at that time point generalizes to these other time points. This in turn suggests that (part of) the underlying cortical signal is stable across this time interval. Distinct patterns in the temporal generalization matrix allow one to draw different conclusions about the dynamics underlying neural processing (for details see King and Dehaene, 2014). In ADAM, the cfg.crossclass parameter specifies whether to compute temporal generalization or not. If cfg.crossclass is set to 'yes', ADAM computes a train × test generalization matrix, which can subsequently be statistically analyzed and visualized at the group level. The diagonal of the train × test performance matrix is the same time series that is computed when cfg.crossclass is set to 'no' (this is because for these diagonal time points, the classifier is trained and tested on the same time points). For this reason, training and testing on the same time points is sometimes referred to as “diagonal decoding.” If cfg.crossclass is set to 'yes' when computing the first level (single subject) results, this affords maximal flexibility when performing group level analysis using adam_compute_group_MVPA (see section 2.11 further below). For example, one can either compute the full train × test temporal generalization matrix at the group level, compute only the diagonal at the group level, or average over particular train or test intervals at the group level (also see section 2.12). However, computing temporal generalization does require much more computing time. To save time, one can opt to have ADAM downsample the input signal prior to first level analysis (see section 2.9.8). If cfg.crossclass is set to 'no' computation time is relatively short, but in this case one can only compute and plot statistics at the group level for the diagonal (training and testing on the same time points).

#### 2.9.8. Pre-processing: channel selection, resampling, baseline-correction

ADAM assumes that input files are already pre-processed (e.g., in Fieldtrip or in EEGLAB), but to make life a little easier ADAM is able to perform some basic pre-processing steps. For the analyses discussed here, no pre-processing was applied to the data prior to ADAM analysis other than epoching and down-sampling. ADAM provides four noteworthy internal pre-processing options: electrode/channel selection, resampling, baseline-correction and muscle artifact rejection.

Channel selection is done using cfg.channelpool. This option makes it possible to select electrodes/channels (these are called the “features” in a decoding analysis) prior to computing classification performance. Although classification algorithms already intrinsically up-weigh features that contribute to classification performance and down-weigh features that do not, sometimes a signal is known to be contained in a particular part of the brain. For example, when using a visual task, occipital channels are likely to be most informative. In such cases, classifier accuracy can be boosted by pre-selecting channels. To keep all channels, use 'ALL_NOSELECTION'. More information about channel selection can be found by typing help adam_MVPA_firstlevel and/or help select_channels in the MATLAB Command window. In the current analysis, no electrode/channel selection was applied.

In addition, it is possible to down sample the signal prior to running an analysis by specifying a new sampling rate using cfg.resample. The main advantage of doing this is to save computation time (at the expense of temporal resolution of course). This is of particular importance when running cross classifications to compute temporal generalization matrices, in which the analysis is performed for every train and test time combination (see section 2.9.7). When performing decoding on TFRs, ADAM will use the original sampling rate to compute TFRs, and only perform decoding on time points that belong to the redefined sampling rate after power has been computed. In the current analysis, the data were resampled to 55 Hz.

Third, a very common step in ERP analysis is to apply baseline correction. ADAM can do this automatically by specifying cfg.erp_baseline (in seconds). In the current analysis, a baseline correction between -100 and 0 ms was applied.

Finally, it is possible to remove trials containing muscle artifacts in a certain window of the trial using the cfg.clean_window parameter. This step was not applied in the current analyses. One can pre-process the data using any personal choice prior to using ADAM, as long as the data are epoched. An overview of the effect of various pre-processing steps on classification performance is given by Grootswagers et al. (2017).

#### 2.9.9. Running the first-level analyses

When running adam_MVPA_firstlevel using the example code at the start of this section, it will classify the activity across all electrodes for each train-test sample in a trial as either coming from a famous face or from a non-famous face, and compute average classification performance for each of these samples. The result of each subject's analysis will be written to disc. The directory to which the first level results are written is specified using cfg.outputdir. The output directory should contain a name that is specific to a given analysis (see example code). If a directory does not exist, ADAM will create that directory. The resulting data structure will be briefly explained in section 2.10. For the current manuscript, we ran three first level analyses for EEG and the same three for MEG, so a total of six first level analyses. The code above already ran the first analysis. Assuming that the variables from that code (containing the event specifications etc.) are still in MATLAB's workspace, it is easy to run each of the remaining five analyses using the code below.

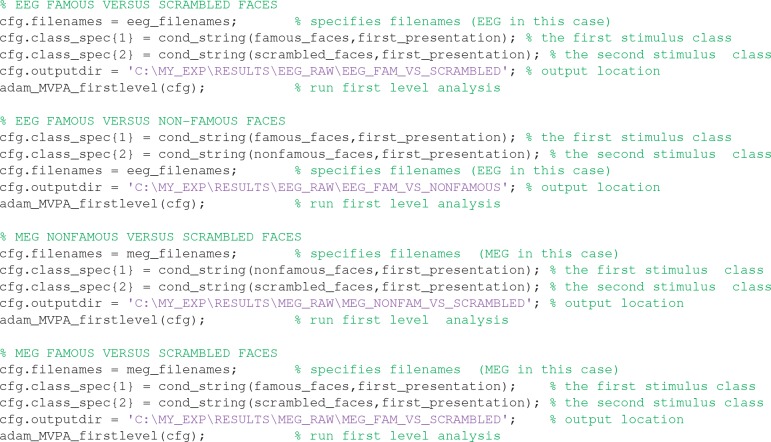

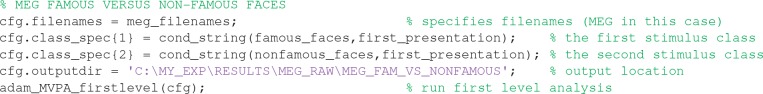





### 2.10. First level folder structure

The results of a first level analysis are stored in a directory path specified in cfg.outputdir. In the example above, the first relevant directory in this path is typically called RESULTS, followed by a directory indicating whether decoding was performed on raw EEG data (EEG_RAW) or raw MEG data (MEG_RAW), but for a time-frequency analysis one might indicate something like EEG_TFR or MEG_TFR. The last folder in the directory structure denotes the comparison in the analysis (e.g., in the example above, decoding famous vs. non-famous faces using EEG data was indicated using EEG_FAM_VS_NONFAMOUS). Inside this folder, ADAM will further automatically create separate folders for analyses based on different channel selections, as specified in cfg.channelpool (see section 2.9.8). If an analysis uses all electrodes/channels as in the current example, this folder will be named ALL_NOSELECTION, but if specifying to only use occipital electrodes, it will create a folder called OCCIP for that analysis. This directory will typically contain .mat data files containing the first level result for each of the individual subjects, or may contain separate folders for each frequency in case it pertains to a decoding analysis of time-frequency data. It is advisable to use a clear and unambiguous naming scheme when specifying cfg.outputdir, as in the example above. If one or more of the directories in cfg.outputdir do not exist, ADAM automatically creates the hierarchy with all the missing directories.

### 2.11. Group analysis workflow

Once the first level analyses have completed, the next step is to perform group analysis and visualization. ADAM has two functions that perform group analysis: adam_compute_group_ERP and adam_compute_group_MVPA. Group statistics on ERPs are computed using adam_compute_group_ERP (ADAM automatically also saves ERPs when running first level analyses), while group statistics on multivariate analysis results are obtained using adam_compute_group_MVPA. Both functions read the results from the first level single subject files that are contained in the RESULTS folder and perform a group analysis on these data. This returns a group stats variable that contains the outcome of one or more analyses (explained in section 2.12 below), which can subsequently be plotted using the adam_plot_MVPA function (explained in section 2.13 below). In keeping with the logic of the adam_MVPA_firstlevel function, both functions have a cfg variable as input. The cfg variable specifies the parameters that can be adjusted when computing group statistics and plotting results. These parameters will be treated in detail in section 3 (Results), so that they are discussed alongside the output that the functions produce.

### 2.12. Group statistics and multiple comparison correction

The adam_compute_group_ functions read in the outcome of first level analyses and compute group statistics on them. They return the result in a stats variable. When executing one of the adam_compute_group_ functions, a folder selection dialog will pop up. This dialog allows the user to select a first level directory from which to compute the group stats variable. One can either select a directory referring to a specific analysis (e.g., EEG_FAM_VS_NONFAMOUS in the current example analysis) or select one directory higher up that contains multiple first level analyses (e.g., RAW_EEG in the current example analysis). When selecting a folder that contains multiple analyses, ADAM will compute group-level results for all the analyses contained in the folder and return the group results of these analyses in a stats array. A number of examples of how this works are supplied in section 3 (Results).

The group statistics are computed by applying *t*-tests across subjects using the metric that was specified during first level analysis (for MVPA the default performance metric is AUC, see section 2.9.5, for ERPs it is μV). The *t*-tests compare this metric to a reference level for each sample (this reference level is 0.5 chance performance in the case of AUC, or either 0 or a cfg-defined reference condition/channel when computing ERPs). ADAM can constrain the range of tests by pre-selecting a train and/or test time window and/or range of frequency bands. In addition, ADAM can average across any of these time windows or frequency ranges. This is particularly relevant when the first level analyses contain time-frequency results (see section 2.9.4) and/or temporal generalization (see section 2.9.7). Examples of how to constrain the time points that are used in a group-level analysis using the cfg variable are given in the results section, as for example in section 3.8.

The outcome of a group analysis yields a *p*-value for every sample. Because large numbers of tests result in the well-known multiple comparison problem (Bennett et al., 2009), ADAM has two ways of controlling for multiple comparisons at the group level. One option is to apply cluster-based permutation testing, in which clusters are defined as contiguously significant *t*-tests. The size of each observed cluster is defined as the sum of the *t*-values in that cluster. Next, this procedure is repeated many times (1000 by default), each time randomizing the condition labels (e.g. the AUC value and its reference .5 value) for each subject prior to performing the *t*-tests. These iterations allow one to compute a null distribution of cluster sizes under random permutation against which to compare the actually observed cluster sizes, based on which the *p*-value for each actually observed cluster can be directly computed (section 5 in Maris and Oostenveld, 2007). This limits the number of hypothesis-related tests to the number of observed clusters, severely limiting the number of relevant statistical comparisons. The standard *p-*value used to delineate whether a given sample is part of a cluster is 0.05. Alternatively, one can apply multiple comparison correction using the False Discovery Rate (FDR) under dependency (Benjamini and Yekutieli, 2001). FDR correction limits the false positive rate q, such that no more than a fixed percentage of tests (usually 5%) of the total number of significant tests can reasonably be expected to be false positives (type I errors). When either correction is applied, only tests that survive the threshold under that correction are plotted as significant by adam_plot_MVPA. Examples of both correction methods are given in the results section, as for example in section 3.1.

It is also possible to directly compare different first level analyses to each other. This is achieved by the adam_compare_MVPA_stats function. In this case, two first level metrics from different analyses are compared against each other using *t*-tests. The same multiple comparison corrections can be applied as in the adam_compute_group_ functions. Note that this is usually only sensible when the data come from the same experiment and/or subjects, as different experiments may have different signal to noise ratios, hampering interpretation. An example of this analysis is given in section 3.7 of the results.

Also note that some caution is in order when drawing population level inferences from statistics computed on MVPA metrics. In particular, standard statistical tests of classification performance against chance do not allow population level inference when the train and test set are drawn from the same distribution (i.e., when the when they both come from the same task), as is the case in a k-fold analysis (see section 2.9.6). In this case, the results should be interpreted as fixed- rather than random effects (see Allefeld et al., 2016 for details). This can be resolved by computing information prevalence across the group, but this metric has not yet been implemented in the current version (V1.0.4) of ADAM. Population level inference is not jeopardized when train and test sets are drawn from different distributions, as when the training data are obtained from a different task than the test data, when evaluating off-diagonal classifier performance in a temporal generalization matrix (see section 2.9.7), or when different first level analyses are compared to each other at a group level (as happens when using the adam_compare_MVPA_stats function).

### 2.13. Plotting group results

Group results are plotted using adam_plot_MVPA. This function requires two inputs: a cfg and one or more stats variables produced by the adam_compute_group_ functions. Each stats variable can contain a single analysis but can also contain multiple analyses in an array (see section 2.12). ADAM either visualizes the outcomes of all analyses that it receives in a single figure or plots them as separate figures. Plotting is always constrained by the settings that were applied when computing the group statistic (see section 2.12). As a result of these settings, the plotting function can visualize two types of graphs: either line graphs that plot classifier performance on the y-axis and time on the x-axis, or graphs that plot classifier performance using a color scale. Color scale graphs either have train-time and test-time on the x- and y-axis (in the case of temporal generalization), or frequency on the y-axis and time on the x-axis (when the first level was performed with time-frequency option). Significant time windows in line graphs are indicated by using a thicker line, which is placed on the line graph itself and/or near the time axis. Significant samples in color graphs are indicated using saturated colors. Unsaturated (bland) colors either reflect *p*-values that do not survive the uncorrected threshold, or are below the multiple-comparison corrected threshold, depending on the settings applied when computing the group-level statistic (section 2.12).

The cfg variable specifies the parameters to adjust the plot, such as tickmarks, y-limits, the order of the plots (in case of multiple analyses), whether to plot the results in a single graph or in multiple graphs (in case of multiple analyses) and so forth. Examples of these options are given in the results section, along with the code that produces the graphs. In addition, the help file of adam_plot_MVPA provides a detailed description of the options.

## 3. Results

### 3.1. ERPs and difference waves of ERPs

In the first group-level analysis, we compute the group results from the first-level analysis of the comparison between non-famous and scrambled faces. We will compute the raw ERPs of non-famous and scrambled faces, and also their difference, and subsequently plot everything in a single plot. First, we will compute the raw ERPs. When running the code below, a selection dialog will pop up from which a folder can be selected. The first-level analyses that will be plotted are contained in the folder EEG_NONFAM_VS_SCRAMBLED (inside EEG_RAW), so that is the folder to select. Because it is cumbersome to have to navigate to the RESULTS folder for every a particular group result, the user can point the function to the root folder for the first-level analyses using cfg.startdir:

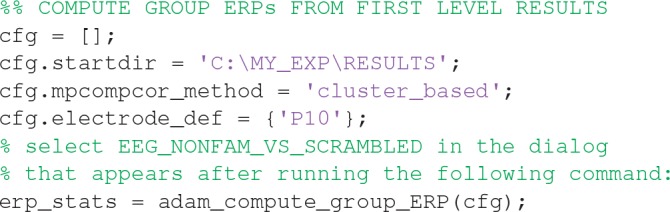



Two other relevant settings are cfg.mpcompcor_method, which specifies the method used to correct for multiple comparisons (cluster based permutation testing in this case, Maris and Oostenveld, 2007), and cfg.electrode_def, which specifies the electrode(s) to obtain ERPs for. The user can also specify the *p*-value cut-off values (default: 0.05) and whether to use one-tailed or two-tailed testing (default: two tailed). More information about these and other settings can be found by inspecting the help of adam_compute_group_ERP. Once the function has finished, the erp_stats variable will contain group ERPs of the classes that were specified when running the first level analysis (the first class contained initial presentations of non-famous faces, the second class contained initial presentations of scrambled faces, see the code in the beginning of section 2.9). Next, to compute the difference between these ERPs, the function needs to be executed once more, this time specifying 'subtract' in cfg.condition_method. When running the code below, the selection dialog will pop-up once more, where the user should select the EEG_NONFAM_VS_SCRAMBLED folder as before.

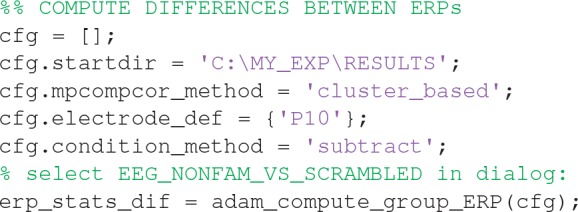



The snippets of code above will now have produced two variables, one called erp_stats (containing the separate ERPs) and one called erp_stats_dif which contains the difference between these ERPs. ERPs and other stats variables can be plotted using adam_plot_MVPA. This function has two inputs. The first input is a cfg variable, specifying the parameters that are relevant to adjust the plot, the second input contains the stats variable containing the data to plot. Two (or more) stats variables can be plotted using a single adam_plot_MVPA command by enumerating them after the cfg variable while separating them using commas:

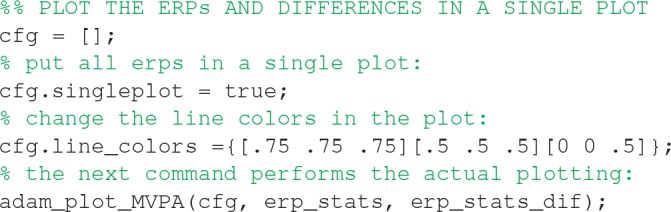



**Figure 2:**
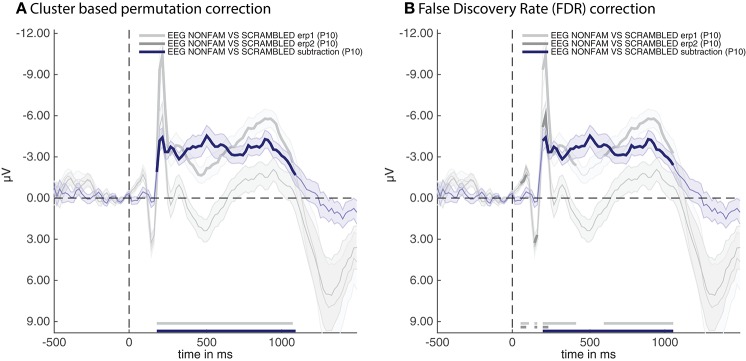
ERPs for the non-familiar (erp1) and scrambled (erp2) condition as well as their difference (subtraction). **(A)** Thick lines denote *p* < 0.05 under two-sided cluster-based permutation (Maris and Oostenveld, 2007) **(B)** Thick lines denote *q* < 0.05 for dependent observations under FDR correction (Benjamini and Yekutieli, 2001).

Two cfg parameters of adam_plot_MVPA are noteworthy here. The first is cfg.singleplot. This setting specifies whether all the analyses in the stats variable are plotted together in a single plot, or whether the function produces a different plot for every stats variable. Try setting cfg.singleplot = false (which is the default) to see the effect. The other is the cfg.line_colors setting. ADAM uses default line colors for graphs, which can be changed using the cfg.line_colors parameter. This parameter specifies the RGB colors of the lines that are plotted using a triplet of values between 0 and 1 for every line to denote the contribution red, green and blue respectively (type doc ColorSpec in the MATLAB Command window for more information). In the plot presented here, the colors were changed to make them consistent with the remaining plots in the results section. The snippet of code above produces the plot shown in Figure 2A.

The thick parts of the line are parts of the time-series that are statistically significant after applying the correction method that was specified when producing the group stats variable, these are also plotted near the time axis at the bottom. The shaded area around the line is the standard error of the mean across participants. Note that the initial C1 and P100 component of the raw ERPs (erp1 and erp2) do not reach significance despite having a very small standard error. This is due to the fact that cluster-based permutation testing robustly determines clusters (including cluster onsets) but is less sensitive to focal regions of significant activity (as would be the case for the peaks of the C1 and P100 components). If one is interested in small windows of highly significant activity, it might be better to apply an FDR correction (Benjamini and Yekutieli, 2001). In the current example analysis this can easily be achieved by re-running the group-level script above after replacing the line that says cfg.mpcompcor_method = 'cluster_based'; with cfg.mpcompcor_method = 'fdr';. Plotting the result again indeed shows that both the C1 and P100 of erp1 and erp2 reach significance under FDR correction (see Figure 2B). However, the disadvantage of FDR correction is that it is less robust to assessing the onset of large clusters (resulting in later onsets than is observed under cluster-based permutation, a similar detrimental effect of FDR correction on cluster onsets can also be seen in Grootswagers et al., 2017, Figure 14), and less robust to identifying sustained clusters (compare the continuous significance of erp1 in Figure 2A to the interrupted significance line of erp1 in Figure 2B). In the remainder of the manuscript we will consistently be using cluster-based permutation testing but alert the reader to the impact of using different types of multiple comparison correction. We also point out that the adam_plot_MVPA function has many parameter settings, allowing one to specify the tick-marks of the x- and y-axis in the graph, inverting the direction of the y-axis (negative up or negative down) etc. These parameter settings will be treated further down or can be found in the help documentation of the adam_plot_MVPA function (type help adam_plot_MVPA in the MATLAB Command window).

### 3.2. Difference waves of ERPs

In the second analysis, we compute the outcome of three ERP subtractions in the experiment: non-famous vs. scrambled faces (as in the first analysis), famous vs. scrambled faces and non-famous vs. famous faces. Below is the code to compute these three group analyses. When running this snippet of code, a selection dialog will pop up. This time, select the EEG_RAW folder (which contains all of these three contrasts, as these were computed in the first level analysis).

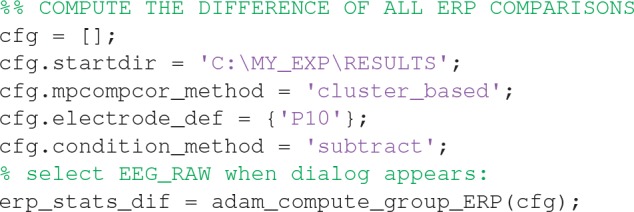



The outcome of this analysis is contained in the erp_stats_dif variable, which is an array containing the outcome of the three analyses.

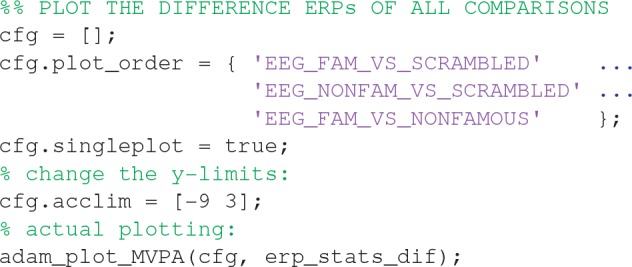



**Figure 3:**
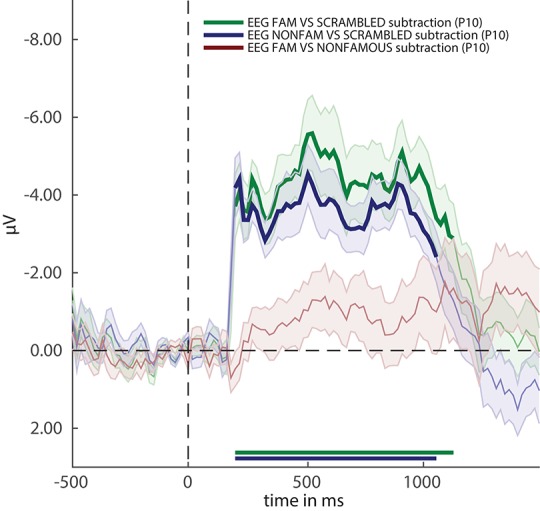
ERP difference waves for the three comparisons in the experiment. Thick lines denote *p* < 0.05 under two-sided cluster-based permutation (Maris and Oostenveld, 2007).

Running the snippet of code above produces the plot shown in Figure 3. Two more cfg parameter settings are introduced here. The first is called cfg.plot_order. This parameter specifies the order in which the comparisons inside the EEG_RAW folder (which was selected when computing group results) are plotted. The plot order impacts the order in which the default colors are used for plotting, and accordingly the order of the names in the legend. When omitting this parameter, the plot function will use the order in the stats variable. Another parameter is the acclim parameter, which sets the bounds for the y-axis in line graphs. When omitting this parameter, the function will use default bounds (which are usually fine). Here we adjusted them slightly to remove overlap between the plots and the legend.

Figure 3 reveals that two out of three ERPs difference waves (subtractions between raw ERPs) result in windows of activity in which the difference is significant (as indicated by thick lines).

### 3.3. Inspecting the stats structure

These temporal windows (their start and stop point in milliseconds and the time at which they peak) can be inspected in the stats structure. For example, to inspect the third stats variable, type:

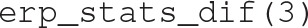



This displays the contents of this analysis in the MATLAB Command window:

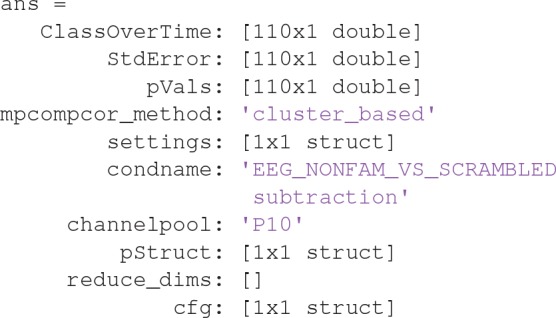



The condname field shows that this is the analysis that compares non-familiar faces to scrambled faces. Other analyses can be inspected by putting a different number between the parentheses. The mapping between the number and the analysis that was performed may differ depending on how the operating system orders files. To enforce a particular order, specify cfg.plot_order when calling adam_compute_group_ERP). The stats structure also contains a field called pStruct. This contains the values of the significant clusters in this analysis. The pStruct field can be accessed by typing:

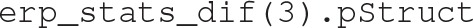



This will show the following in the Command window:

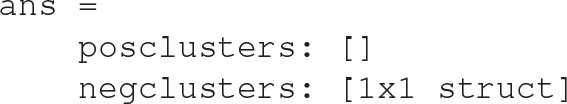



The pStruct field contains two fields, one for positive clusters and one for negative clusters. As can be seen from Figure 3, the significant window for EEG_NONFAM_VS_SCRAMBLED is negative (by convention negative is often plotted upwards when plotting ERPs), and indeed the posclusters field is empty, as it is followed by empty square brackets []. To inspect the negative clusters, type:

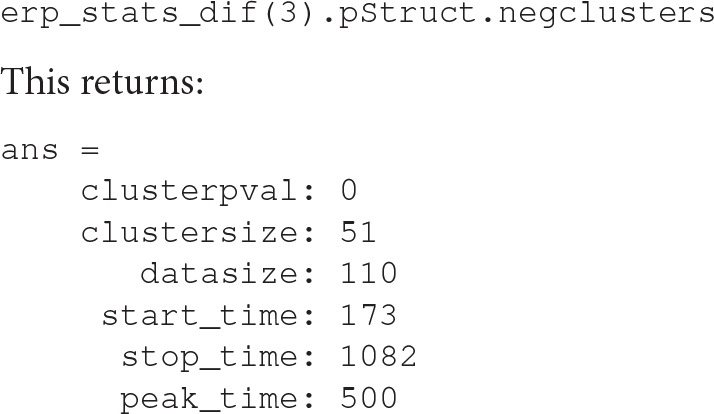



The first field is called clusterpval. This is the cluster based p-value after cluster based random permutation (Maris and Oostenveld, 2007). In this case, the value is 0. By default, the cluster-based permutation test in the adam_compute_group_ functions run 1,000 times. The fact that the clusterpval is 0 means that a cluster of the actually observed size was never obtained under random permutation, so that the *p*-value under permutation is smaller than 1/1000, hence this *p*-value should thus be reported as *p* < 0.001. The clustersize field reflects the number of consecutive samples in the time window, the datasize field reflects the total number of samples in the time series, the start_time reflects the onset time in milliseconds of the significant window, the stop_time the offset time in milliseconds, and the peak_time reflects the time point at which the ERP difference was maximal. The same information can also be obtained for decoding analyses, e.g., by inspecting the stats structure that results from running adam_compute_group_MVPA instead of adam_compute_group_ERP.

### 3.4. Training and testing on the same time points (diagonal decoding)

Next, we cover how to apply a decoding analysis using very similar code as was used to compute ERPs. First, we will run the equivalent of the ERP analyses that were computed in the previous sections, this time using adam_compute_group_MVPA. When running the following code, a selection dialog will pop up again. Select the RAW_EEG folder, after which the group analyses will be performed.

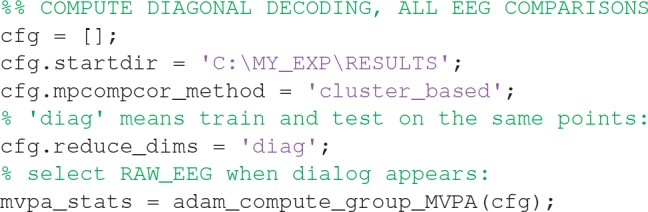



After running the code above, the decoding results for all analyses contained in the RAW_EEG folder will be contained in the mvpa_stats variable. The only new setting in the above is the cfg.reduce_dims variable. For now, it is sufficient to remember that setting this to 'diag' means that this extracts the decoding analysis in which the classifier was trained and tested on the same points (so without looking at temporal generalization, see section 2.9.7). To plot the decoding results, again very similar code is used as before:

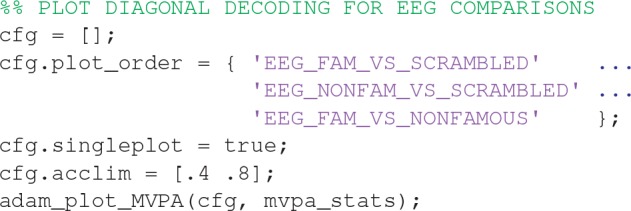



**Figure 4:**
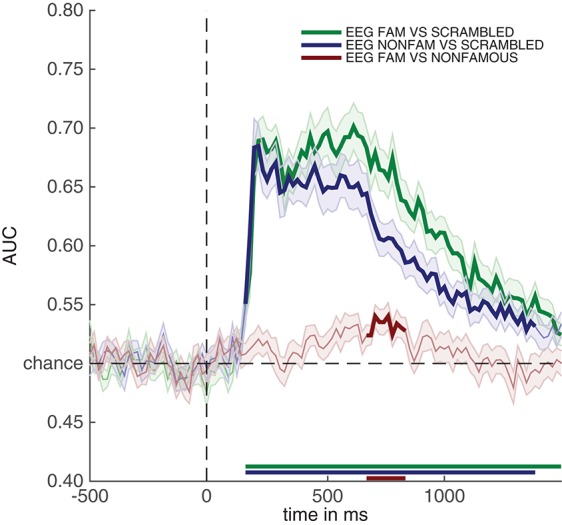
Classification performance across time for the three comparisons in the experiment. Thick lines denote *p* < 0.05 under two-sided cluster-based permutation (Maris and Oostenveld, 2007).

Running the snippet of code above produces the plot contained in Figure 4. This figure looks comparable to the plot contained in Figure 3, but this time the y-axis denotes classification performance (rather than μV as in the ERP analyses). As can be seen from Figure 4—and perhaps unsurprisingly - decoding produces somewhat similar results as the ERPs in Figure 3. Two out of three decoding results show windows of activity in which accuracy is significant after correcting for multiple comparisons using cluster-based permutation (as indicated by the thick lines). However, there are also some notable differences. For example, decoding of famous vs. scrambled faces is significant for a longer period of time than the ERP subtraction of this comparison at electrode P10. Moreover, the difference between famous and non-famous faces never reaches significance in the ERP, but does reach significance in the decoding analysis. Both differences between decoding results and the ERP at the P10 electrode must be due to the fact that there is information contained in the multivariate pattern of activity across the scalp, which exceeds the information that is contained in the P10 electrode alone. This demonstrates one of the strengths of the decoding technique: MVPA allows one to obtain a measure for the difference between two conditions (stimulus classes) without having to specify a priori in which electrode this difference emerges, while at the same time picking up subtle differences that might not have been noticed had such an a priori electrode selection been made, also see (Fahrenfort et al., 2017a). In section 3.6 further below we explain how to visualize the pattern of activity that underlies classification performance using topographical maps.

### 3.5. Plotting single subject results

A nice feature of ADAM is that it allows quick visualization of group results (ERPs, classification performance etc.). However, it is unwise to simply compute a group result without also inspecting single subject results. For example, one should typically ascertain whether the group result was caused by only a few participants or whether the effect is present in most of the participants in the sample (Allefeld et al., 2016). Moreover, it may be that some participants show irregularities, for example due to incidental equipment failure, software bugs, or bad signal to noise ratio. It requires only a single line of code to also display single subject results when computing group results, by setting cfg.plotsubjects to true. In the code below, the single subject results for an analysis are plotted. When running the code, select EEG_FAM_VS_SCRAMBLED in the RAW_EEG folder when the selection dialog pops up.

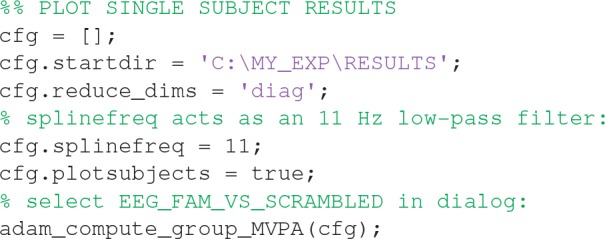



**Figure 5:**
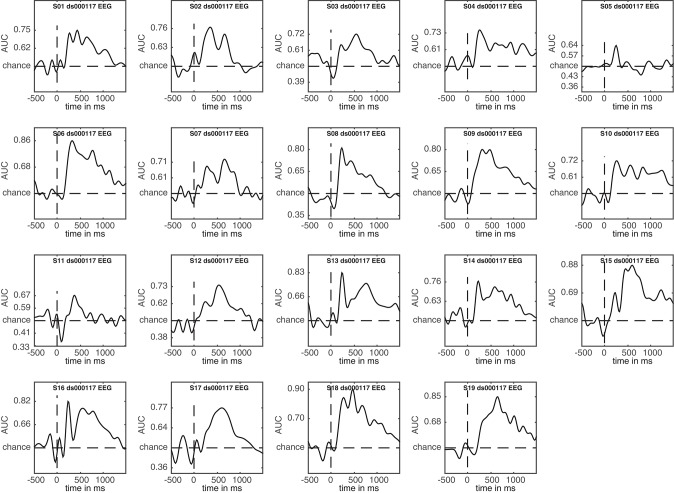
Decoding results of individual subjects for the famous vs. scrambled faces comparison.

The result is shown in Figure 5. This figure shows the first level decoding result when comparing famous to scrambled faces. Single subject results are displayed on a grid, with the vertical axes equalized to enable easy comparison. The tick-marks are set on half the maximum classification performance of that subject. This way, one can quickly inspect whether all subjects show approximately the same effect, or whether any subjects show large deviations. Note that the code also specifies a splinefreq parameter in the cfg variable. When specifying this parameter, the data is down-sampled to that frequency, always including the sample that contains the largest peak (or trough) in the data. Subsequently, a spline is fitted through this down-sampled signal. This procedure effectively acts as a low-pass filter that retains the maximum (or minimum) in the signal, while removing high frequency information. This parameter is particularly useful when the results contain lots of high-frequency noise (as is typically the case for individual subjects) and is only applied as a visualization step. Statistical testing is always applied to the unaltered data. The cfg.splinefreq parameter can of course also be applied when plotting at the group level, although we chose not to do so here.

### 3.6. Topographic maps

As mentioned before, it is often useful to know the pattern of neural activity that gives rise to classification performance. However, weight vectors (the weights that correspond to the features resulting from the training procedure in a decoding analysis, electrodes in this case) are not directly interpretable as neural sources (Haufe et al., 2014). Therefore, ADAM can transform the weight vectors from BDM analyses to weights that would result from a forward model. The procedure for this transformation is simple, and results in activation patterns that are directly interpretable as neural sources, thus allowing one to plot an interpretable topographical map of the activity that underlies the decoding result. The transformation has previously been described by Haufe et al. (2014), and involves taking the product of the classifier weights and the data covariance matrix. The resulting activation patterns are equivalent to the topographical map one would obtain from the univariate difference between the stimulus classes that were entered into the analysis. Yet, it is slightly more elegant to derive them this way because of the direct mapping between the decoding analysis and the topographical maps (at the same time providing a sanity check of the data integrity of the analysis).

Alternatively, one can visualize the correlation/class separability maps that are obtained by taking the product of the classifier weights and the data correlation (instead of covariance) matrix. Correlation/class separability maps visualize activity patterns for which the task-related signal is both strong and highly correlated with the task, while at the same time minimizing the influence of strong artifacts such as eye-blinks (Haufe et al., 2014; Fahrenfort et al., 2017b). The following code visualizes the activation patterns resulting from the product of the forward transformed decoding weights topographical maps, for each of the three main analyses.

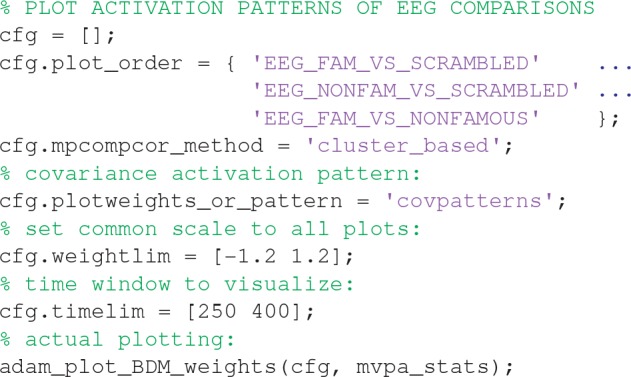



**Figure 6:**
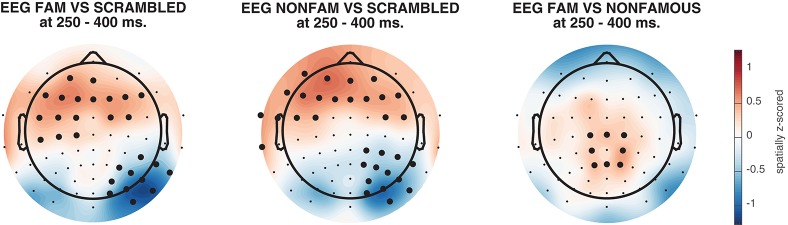
Activation patterns from 250 to 400 ms, spatially normalized (z-scored) for every subject. Thick electrodes denote *p* < 0.05 under two-sided cluster-based permutation (Maris and Oostenveld, 2007).

The resulting topographical maps can be found in Figure 6. Interestingly, all three comparisons show clearly significant clusters after cluster-based permutation (Maris and Oostenveld, 2007). This is especially surprising for the famous vs. non-famous comparison, as classification performance was not significantly above chance for this comparison in this time interval (see Figure 4). This could have a number of causes. For example, in Figure 6 we plot topomaps for a particular time window (average between 250 and 400 ms), rather than looking at above chance classification performance across time, so the pre-selection of a temporal window is likely to impact the outcome of the cluster-based test. Relatedly, Figure 6 shows a cluster-based permutation test across electrodes (looking for clusters of contiguous electrodes that remain significant after random permutation), whereas Figure 4 performs a cluster-based permutation test of classification performance across time (looking for clusters of contiguous time samples that remain significant after random permutation). Finally, it is important to realize that a given classifier may not always succeed in extracting the relevant features to achieve above chance classification performance, even when there is potentially relevant information in the data. Selecting a subset of features (electrodes/channels), a different accuracy measure (Bradley, 1997), different pre-processing steps (Grootswagers et al., 2017), or a different train-test algorithm (Cox and Savoy, 2003; Grootswagers et al., 2017) may all impact the degree to which a decoding analysis yields above chance classification performance.

### 3.7. EEG and MEG temporal generalization results

An additional advantage of performing multivariate analysis over ERPs is the ability compute the stability of neural activity over time by inspecting the so-called temporal generalization matrix (King and Dehaene, 2014). Temporal generalization matrices display how well classification performance for a given time sample generalizes to all other time samples. Thus, a classifier is trained for every sample, and each of these classifiers is tested on all samples in the trial. If a classifier that was trained on a given sample yields high classification performance across samples from all other time points, this shows that the neural pattern of activation is stable, otherwise classification performance would not generalize to these other samples. The ability to inspect temporal generalization matrices needs to be specified during first-level analysis by setting cfg.crossclass = 'yes' (which was indeed the case, see section 2.9.7).

In this section, we compute temporal generalization matrices for all three comparisons, separately for the EEG data and for the MEG data. When running the code below, a selection dialog will appear twice. The first time it appears, one should select the EEG_RAW folder, the second time it appears, one should select the MEG_RAW folder.

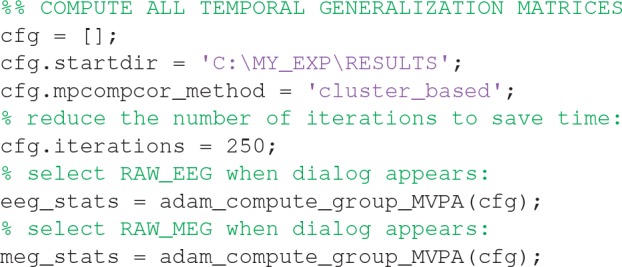



The results of the EEG and MEG temporal generalization matrices are now stored in eeg_stats and meg_stats respectively. Importantly, we did not specify cfg.reduce_dims here, as we did when we previously ran adam_compute_group_MVPA. This means that the group analysis is applied to the entire temporal generalization matrix that was computed during the first level analyses. Another thing to note is that we specified cfg.iterations = 250. This lowers the number of iterations that the cluster-based permutation test applies to 250 iterations, rather than the default 1000 iterations. This is merely done to save some computation time; with the only implication that the obtained *p*-values are slightly less accurate. To obtain more accurate cluster-based *p*-values keep the default at 1000 or higher. To plot all resulting group temporal generalization matrices, both for EEG and MEG, run the code below.

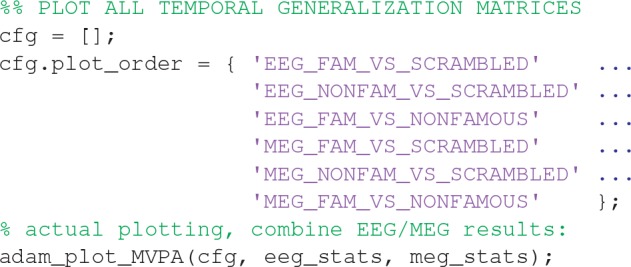



**Figure 7:**
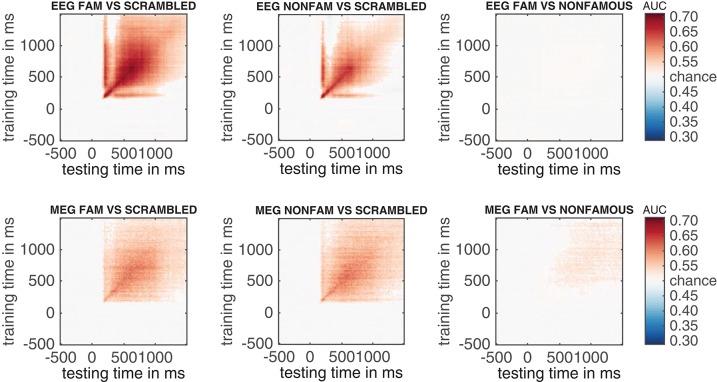
Temporal generalization plots for all six analyses. The plots show the degree to which the classifier when trained on a given time point (on the y-axis), generalizes to other time points in the trial (on the x-axis). Color indicates classifier performance using AUC. The diagonal (from the left bottom to the top right) shows classification performance when the classifier is trained and tested on the same time point. More off-diagonal activity indicates stronger temporal generalization. (top row: EEG, bottom row: MEG).

The result can be seen in Figure 7. This figure shows the temporal generalization matrices for all three EEG comparisons in the top and for MEG in the bottom row. The eeg_stats and meg_stats variables passed as a comma separated list as before, and the cfg.plot_order parameter specifies the order in which to plot the comparisons, as has also been shown previously. When eyeballing these graphs, there are three notable differences between the EEG and MEG results. The first is the fact that the EEG matrices seem to achieve higher classification performance in the faces vs. scrambled comparisons when compared to MEG, especially along the diagonal where the result is darker red for EEG than for MEG. The second is the observation that the MEG seems to show better temporal generalization than EEG, as the colored portion of the MEG graphs extends further away from the diagonal (i.e., is more “square”) than that for the EEG graphs.

The third notable observation is that the famous vs. non-famous graph shows significant differences in MEG, but not in EEG.

### 3.8. EEG and MEG stability over time when training on 250–400 ms

To understand and visualize these differences more easily, it can be advantageous to pick a training time window and investigate to what extent that window generalizes to other time samples in the trial. For illustrative purposes, we use a training window between 250 and 400 ms, and plot how well the neural pattern observed in that window generalizes to the rest of the trial. When running the code below, as in the previous section, first select the EEG_RAW folder, and then the MEG_RAW folder.

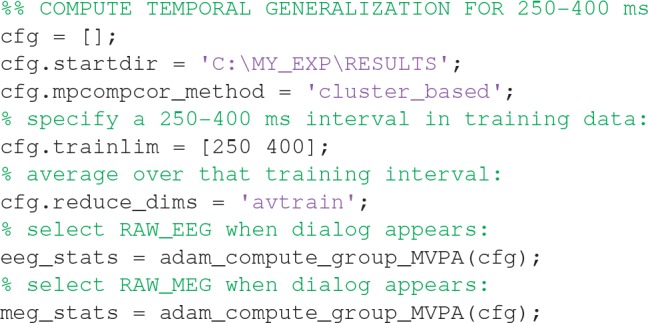



Two new cfg parameters are important here: cfg.trainlim and cfg.reduce_dims. The trainlim parameter specifies the temporal window in milliseconds to which the training data (vertical axis in Figure 7) should be limited. The parameter cfg.reduce_dims = 'avtrain' averages over the training window, in this case the period between 250 and 400 ms. The resulting stats structures evaluate how that train window generalizes to all other samples in the trial. This can subsequently be plotted using:

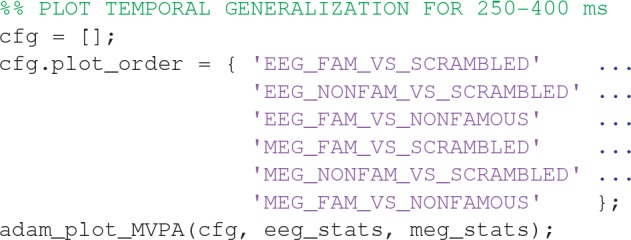



**Figure 8:**
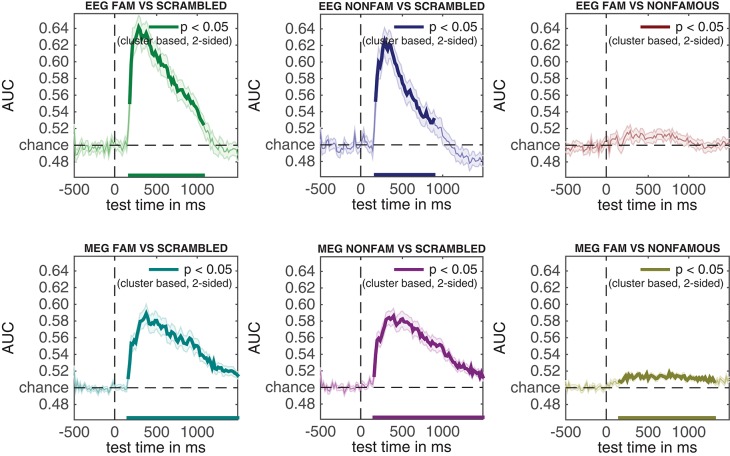
Assessing cortical stability in EEG and MEG through generalization across time given a 250–400 ms training window.

This produces the line graphs in Figure 8, which again show EEG in the top row and MEG in the bottom row. If decoding stays high throughout a line graph, this shows that the neural pattern of cortical activity that occurs between 250-400 ms is stable over time, as it is able to drive classification performance at all other time points. As one can see in Figure 8, this is indeed the case for MEG, where classification performance remains above chance all the way to the end of the trial at 1,500 ms. However, this is not the case for EEG, where classification performance drops off to chance toward the end of the trial period (in the faces vs. scrambled comparisons) or is at chance altogether (in the famous vs. non-famous faces comparison). This seems to confirm the observation that was made in Figure 7 that face-related processing generalizes better in MEG than in EEG. Also confirmed are the observations that initial decoding seems higher for EEG than for MEG and that classification performance for famous faces vs. non-famous faces is only significant for MEG and not for EEG.

### 3.9. Comparing EEG and MEG decoding accuracies directly

Although seemingly interesting, the differences between EEG and MEG so far have been established by observing significance in one comparison while not observing significance in another comparison and/or eye-balling the data. For example, the famous faces vs. non-famous faces comparison yields significance in MEG, but not in EEG. However, such observations do not allow one to infer that EEG and MEG are differentially sensitive to the famous faces vs. non-famous faces comparison. That inference would require an explicit statistical test (Nieuwenhuis et al., 2011). As long as the data come from the same experiment and the same subjects—decoding analyses provide a common dependent measure to compare the extent to which different methodologies are able to recover differences between experimental conditions. To formally evaluate differences in classification performance across time between MEG and EEG, they can be compared in a statistical test. The adam_compare_MVPA_stats function provides this functionality. Below the code to directly compare the MEG and EEG stats:

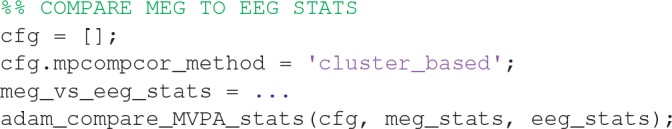



This difference stats variable can subsequently be plotted using the adam_plot_MVPA function as we have been doing all along:

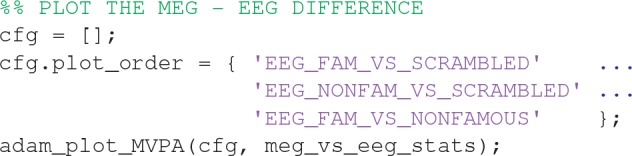



**Figure 9:**
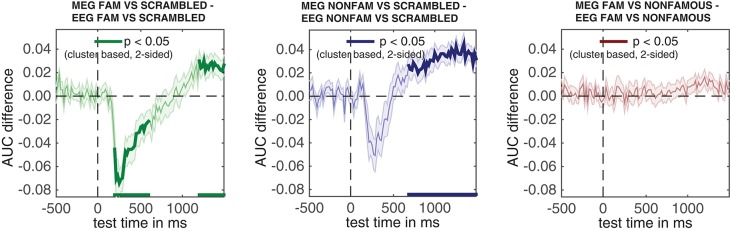
Directly testing the difference in temporal generalization between MEG and EEG given the 250–400 ms training window.

Interestingly, Figure 9 confirms that initial classification performance during the encoding phase is significantly higher in EEG than in MEG during the famous vs. scrambled faces comparison (left graph, below chance classification performance early on), while temporal generalization is significantly higher in MEG than in EEG (left graph, above chance classification performance toward the end of the trial). The same pattern can be seen in the non-famous vs. scrambled faces comparison, although the difference in the initial encoding phase does not survive multiple comparisons correction when applying cluster-based permutation. Although the MEG comparison of famous vs. non-famous faces was selectively significant in the original analysis, the direct comparison between EEG and MEG is not significant, plausibly due to a lack of power.

## 4. Discussion

In this article, we have shown how to analyze a publicly available dataset from Wakeman and Henson (2015) using ADAM. The analysis pipeline described here can easily be ported to other datasets by replacing the input filenames in the script and modifying the class definitions using one's own event codes. In the dataset we analyzed, subjects viewed famous, non-famous and scrambled faces. Unsurprisingly, the results show that ERPs can show similar outcomes as decoding analyses, as long as one knows which electrode(s) to select. However, there are a number of notable advantages to MVPA when compared to standard ERP analysis.

For example, MVPA does not require one to select electrodes, as the decoding analysis automatically extracts informational content from the distribution of activity across all electrodes. Although prior feature (electrode) selection can still be beneficial to improve classification performance (for example selecting only occipital electrodes when a given task is visual), in principle this step is covered automatically by the training phase of a decoding analysis. In the analyses described here, the superiority of this approach becomes apparent when comparing Figure 3 (ERPs) to Figure 4 (classification performance). The decoding graph uncovers a significant difference between famous and non-famous faces that the ERP analysis does not identify. The plausible reason is that the decoding analysis automatically extracts information relevant to the difference between these conditions, which in ERPs would require prior knowledge about which electrodes to select, or require some split half procedure (Kriegeskorte et al., 2009). Of course, this information is also present in the univariate ERPs somewhere (or the classification algorithm could not pick up on it), but experimental differences can be much harder to identify or substantiate using traditional ERPs than using MVPA if the locus of the effect is unknown (also see Fahrenfort et al., 2017a).

Another advantage is that decoding analyses allow one to look at the stability of neural activation patterns over time (King and Dehaene, 2014). This advantage is unique to MVPA, as only multivariate analysis allows one to statistically characterize *patterns* of neural activity. For example, the temporal generalization matrices in Figure 7 reveal the degree to which representations reflecting the encoding of famous and non-famous faces generalize to later time points in a trial. Given the extent of above chance decoding in the far corners of these graphs (the “squareness” of the red-colored region showing above chance decoding performance), these figures suggest that representations of faces during encoding generalize better to other time points when characterizing them using MEG than EEG activity. This suggests that EEG and MEG measurements may be differentially sensitive to stable representations (maintenance) in the face processing network.

To further investigate this, we looked at temporal generalization for a specific time window (between 250 and 400 ms), and subsequently compared this temporal generalization signal between MEG and EEG directly in Figure 9. These graphs reveal that decoding accuracy is better in EEG than in MEG during an early encoding phase, but that MEG generalizes better to points later in time in MEG than in EEG. This interaction in the temporal domain suggests that EEG and MEG tap into different properties of the face processing network: EEG seems to have a higher signal to noise ratio during the fleeting encoding phase, whereas MEG taps into cortical activity that is stable over time, plausibly reflecting maintenance involved in evaluating faces. Together, these analyses reveal a third potential advantage of MVPA. MVPA provides a common measure to directly compare observations obtained from different methodologies, as long as the data are obtained from the same subjects, using the same tasks. In the current manuscript, this was done when comparing EEG decoding accuracies to MEG decoding accuracies, but this methodology in principle also allows one to directly compare neural decoding sensitivity to behavioral sensitivity, as long as the data comes from the same subjects and/or care is taken to properly normalize different dependent measures (Fahrenfort et al., 2017b).

The analysis pipeline described in this article highlights three advantages of MVPA over traditional univariate analysis. A more in depth treatment of the differences between standard univariate approaches and multivariate analysis can be found in Hebart and Baker (2017).

In addition, there are a number of advantages of using ADAM to perform these analyses. ADAM makes it easy to move from ERP, ERF, or TFR-centered research to MVPA analyses, as it enables an easy side-by-side comparison between univariate and multivariate methodologies. This may be particularly helpful for those who have been performing ERP analyses and want to transition to MVPA-centered approaches. ADAM takes EEGLAB or Fieldtrip as input formats, making the switch relatively easy for those who have already been using standard MATLAB analysis toolboxes. To further enable this transition, ADAM takes care of a number of potential confounds that can easily plague an analysis pipeline put together by those not aware of some of the issues. For example, ADAM trades versatility for usability by automatically enforcing balanced designs and by computing AUC rather than overall accuracy. In addition, it allows one to run a multivariate analysis on raw data or automatically perform time-frequency analysis prior to multivariate analysis (not covered in this article), and it easily applies a FEM in addition to a BDM (not covered in this article). Many options are automatically applied by default, or can easily be executed or changed by specifying just one or two parameters in the cfg variable.

Using ADAM also has disadvantages. ADAM is mostly maintained by a single person (the first author of this paper), and for that reason support is limited. ADAM's core functions were initially developed to support standard analyses by the first author, and only later converted into a toolbox to support researchers that are considering a transition from ERP to MVPA analyses. Thus although it aligns with the growing movement to promote open source in cognitive neuroscience (Gleeson et al., 2017), it does not necessarily provide the latest and greatest in multivariate analysis. For those already comfortable with programming and/or multivariate analysis, a number of more versatile alternatives for time-series based MVPA exist which have larger development teams, notably CoSMoMVPA (http://www.cosmomvpa.org, MATLAB) (Oosterhof et al., 2016), the Neural Decoding Toolbox (http://www.readout.info, MATLAB) (Meyers, 2013), the Decision Decoding Toolbox (http://ddtbox.github.io/DDTBOX, MATLAB) (Bode et al., 2018), MNE (http://www.martinos.org/mne/stable/manual/decoding.html, Python) (Gramfort et al., 2014) and the PyMVPA toolbox (http://www.pymvpa.org, Python) (Hanke et al., 2009). Yet, for those wanting to dip their toes into multivariate waters for the first time, ADAM could be a great start.

## Ethics statement

The study was approved by Cambridge University Psychological Ethics Committee. Written informed consent was obtained from each participant prior to and following each phase of the experiment. Participants also gave separate written consent for their anonymized data to be freely available on the internet.

## Author contributions

JF wrote the toolbox, JvD and JF designed analyses, JvD provided import scripts for original data and improved help files for toolbox, JF, JvD, SvG, and CO wrote paper and/or provided editorial guidance.

### Conflict of interest statement

The authors declare that the research was conducted in the absence of any commercial or financial relationships that could be construed as a potential conflict of interest.
